# Health-related personal control predicts depression symptoms and quality of life but not health behaviour following coronary artery bypass graft surgery

**DOI:** 10.1007/s10865-015-9677-7

**Published:** 2015-09-04

**Authors:** Tara Kidd, Lydia Poole, Elizabeth Leigh, Amy Ronaldson, Marjan Jahangiri, Andrew Steptoe

**Affiliations:** Psychobiology Unit, Department of Epidemiology and Public Health, University College London, 1-19 Torrington Place, London, WC1E 6BT UK; Department of Cardiac Surgery, St. George’s Hospital, University of London, Blackshaw Road, London, SW17 0QT UK

**Keywords:** CABG, Control beliefs, Health behaviour, Depression, QOL

## Abstract

To determine the prospective association between health-related control beliefs, quality of life (QOL), depression symptoms, and health behaviours in coronary artery bypass graft (CABG) patients 6–8 weeks following surgery. 149 patients who were undergoing planned CABG surgery were recruited. Patients completed questionnaires measuring health related personal control, treatment control, depression symptoms, QOL, and health behaviours prior to and 6–8 weeks after surgery. Higher levels of health-related personal control predicted better QOL, and lower levels of depression symptoms, but not adherence to medication, cardiac rehabilitation attendance, or physical activity. These results were independent of demographic, behavioural, and clinical covariates. Treatment control was not associated with any outcome. These results suggest that perceived health-related personal control is associated with key aspects of short-term recovery from CABG surgery. Targeted interventions aimed at improving perceptions of health-related personal control may improve health outcomes in this cardiac population.

## Introduction

Coronary artery bypass graft (CABG) surgery is an effective intervention for advanced coronary heart disease (CHD) with over 25,000 surgeries performed in the UK each year and a 98 % survival rate in the months immediately following surgery (Townsend et al., 2012). It is widely accepted that CABG surgery can prolong life, reduce disability, and physical symptoms for those with CHD (Lindsay et al., 2000; Pocock et al., 1996). Despite this, many studies have shown that a significant proportion of patients experience poor recovery following surgery (Blumenthal et al., 2003; Burg et al. 2003). It is believed that a variety of factors are important when trying to understand the disparity in recovery. In particular, there has been growing recognition of the importance of psychosocial factors when explaining variation in recovery following cardiac surgery (Blumenthal et al., 2003; Cserép et al., 2010; Doering et al., 2005).

It has been estimated that at least 25 % of patients report depression, and impaired quality of life (QOL) following CABG surgery (Blumenthal et al., 2003; Burg et al., 2003; Mallik et al., 2005). Moreover, a two-fold increased risk for future cardiac events and mortality 2–5 years following CABG surgery is linked to higher levels of depression (Blumenthal et al., 2003). In addition, patients who experience depression following CABG surgery often suffer impaired QOL, and exhibit low adherence with medical and lifestyle health regimes (Gallagher & McKinley, 2009; Hunt et al., 2000; Oxlad & Wade, 2008).

Improvement in QOL is currently viewed as one of the primary indications of surgery success (Hillis et al., 2011). In addition to being an important predictor of mortality and re-hospitalisation, it is an indication of the emotional and physical well-being of the patient (Mommersteeg et al., 2009; Spertus et al., 2002). While QOL is often used as an indicator of CABG outcome success, engaging in preventive health behaviour are also vital contributors in the recovery process for CABG patients, and may contribute to symptom management and delay of further disease progression (Moser & Dracup, 1995; Poole et al., 2014a). At present, much of this research has focused on long-term outcomes, despite recent evidence suggesting that short-term outcomes are also important in recovery (Kidd et al., 2014; Ronaldson et al., 2014).

Perceived control may have a beneficial effect on mood, QOL, and health-related behaviours in cardiac populations (Barry et al., 2006; Moser & Dracup, 1995; Dracup et al., 2003). Perceptions of control appear to be particularly salient in emotional distress outcomes in cardiac patients, leading to the suggestion that perceptions of control may moderate the effects of depression on health outcomes in cardiac patients (Gallagher & McKinley, 2009). However, this literature has been criticized for not examining perceptions of control specific to cardiac health (Dreher, 2004). Although scant, research suggests that health specific control measures better predict recovery outcomes than those measuring general control (Barry et al., 2006).

Research on the common-sense model of illness has demonstrated that the way that patients make sense of their illness can strongly influence recovery in cardiac patients (Broadbent et al., 2009). Patient beliefs and concerns about the illness have been linked to health behaviours, such as cardiac rehabilitation attendance in myocardial infarction patients (French et al., 2006) and physical recovery indices such as length of intensive care stay in CABG patients (Poole et al., 2014b). Focusing specifically on patient beliefs and expectations about control in relation to their illness and treatment may be a fruitful and innovative approach in explaining variations in depression symptoms and QOL adaptation to illness and recovery.

Health-related control beliefs are made up of (1) how susceptible the illness is to the personal control of the patient and (2) how well it can be controlled by treatment. Patients’ beliefs about treatment are likely to change over time as a function of the treatment received and their subsequent health status (Foxwell et al., 2013). By contrast, perceived personal control over the illness may reflect a more stable underlying belief regarding the extent to which the person is able to control or influence outcomes (Rothbaum et al., 1982). Health-related control beliefs may be a valuable resource not only as an aid in understanding differences in recovery from cardiac surgery, but as an intervention to promote recovery (Petrie et al., 2002).

A recent systematic review of CHD patients found associations between low perceived personal control over their illness and poor QOL. Lower levels of perceived personal control were also associated with higher levels of depression symptoms (Foxwell et al., 2013). Currently, the only prospective study examining illness beliefs and recovery in cardiac patients was conducted by Juergens et al. (2010), who found that pre-surgery personal control was not correlated with depression symptoms, disability, or QOL in patients 3 months after cardiac surgery. However, only 42 patients provided post-surgery data and health behaviours were not included as outcomes in this study (Juergens et al., 2010). Therefore, we decided to conduct analyses examining health beliefs relating to perceptions of personal control and treatment control on markers of post-operative recovery in CABG patients. Based on the existing evidence we surmised that having low health-related control beliefs would be associated with poorer recovery.

## Method

### Participants

The analyses were carried out on patients in the Adjustment and Recovery after Cardiac Surgery (ARCS) Study, a longitudinal study of socioeconomic, psychosocial, and biological risk factors for recovery after CABG surgery. 457 patients were eligible to take part, 340 were consented, but due to withdrawals, change in circumstance, invalid, or incomplete baseline questionnaires, a study sample of 265 CABG patients remained. Patients were recruited at a pre-surgery assessment clinic from a UK hospital on average 29 days prior to surgery. Baseline questionnaires were administered and cognitive function was assessed during this clinic visit. Follow up data was collected at 6–8 weeks following surgery from 215 patients; 2 patients had died, 13 had formally withdrawn, 35 did not respond to the follow up questionnaires. Inclusion criteria for this study were that patients had fully completed the relevant questionnaires at baseline and follow up at 3 months (n = 186), had complete covariate information (n = 160), scored 19 or above on the cognitive function assessment (the Montreal Cognitive Assessment, MoCA), and were undergoing elective CABG surgery (±valve). This left 149 participants with complete data who were eligible to take part in the study (132 males, 17 females, age range 44–90 years). For more information on recruitment, please see Poole et al. (2014b). All procedures were carried out with the written consent of the participants. Ethical approval was obtained from the National Research Ethics Service.

### Measures

#### Clinical and sociodemographic measures

Cardiovascular history and clinical factors were obtained from clinical notes. Clinical risk was assessed using the European System for Cardiac Operative Risk Evaluation (euroSCORE) (Nashef et al., 1999). EuroSCORE is a composite measure of procedural mortality risk based on 17 factors comprising patient-related factors (e.g. age, sex), cardiac-related factors (e.g. unstable angina, recent MI) and surgery-related factors (e.g. surgery on thoracic aorta). Participants detailed their medications prior to surgery and any longstanding illnesses. Socioeconomic status was assessed using yearly household income in five categories ranging from <£10,000 per year to >£40,000 per year. Body mass index (BMI) was assessed at the pre-operative clinic appointment and calculated using the standard formula (kg/m^2^).

### Questionnaires

#### Health-related control beliefs

Patients’ perception of personal control over their illness and treatment were measured using the items related to perceived personal control and treatment control from the Brief Illness Perceptions Questionnaire (Brief-IPQ) (Broadbent et al., 2006). This scale was developed by forming one question that best summarised each subscale of the original IPQ. Items are rated using a 0–10 scale. Although the full Brief-IPQ was administered, for the purposes of this study we are reporting on the two IPQ items relating to perceived personal control and treatment control only. A high score indicates feeling high levels of perceived personal control over the illness and that the treatment will control the illness.

### Psychosocial functioning

#### Quality of life

The Short Form health survey—12-item (SF-12) (Ware et al., 1996) was used in this study at baseline and follow-up as a brief measure of mental and physical QoL. The SF-12 is comparable to the SF-36 in detecting change across time in longitudinal studies, for both the mental and physical component scores (Jenkinson et al., 1997). Responses to the questions were calibrated to give mental and physical component QoL scores with normative values of 50; scores below 50 are suggestive of poor QOL. QOL was assessed at baseline and follow up.

#### Depression

Depression was measured using the Beck Depression Inventory (Beck & Steer, 1987) and assessed at baseline and follow up. The BDI is a 21 item multiple response measure and examines somatic and non-somatic symptoms of depression. The scores vary from 0 to 63 points, and patients with scores lower than 10 can be regarded as asymptomatic in the general population. Higher scores indicate higher distress.

#### Cognitive function

Participants completed an interviewer administered cognitive function test at pre-assessment (MoCA) (Nasreddine et al., 2005). Three measures were selected to assess memory and executive function, as these are susceptible to decline with aging. These included orientation in time, immediate recall, and verbal fluency. Higher scores indicate higher cognitive functioning.

### Health behaviour measures

The International Physical Activity Questionnaire (IPAQ) (Hallal & Victora, 2004) was used to examine the amount of physical activity participants engaged in over the past 7 days. It includes questions about activities at work, house-work, getting from place to place, and activities during leisure time for recreation, exercise or sport. Higher scores indicate greater levels of activity.

Adherence to medication was assessed using a 5 item self-report scale adapted from validated questionnaires (Horne & Weinman, 1999). This is a 5 item self-report scale where participants rate their agreement using a 5-point likert scale. Scores were summed and reversed scored to give a total adherence score ranging from 0 (good adherence) to 20 (poor adherence). Participants were asked if they had attended cardiac rehabilitation at follow up. Answers were indicated by a yes/no response.

### Statistical analysis

Statistical analysis was carried out using SPSS v.20 for Windows. Personal control and treatment control were treated as independent continuous variables. To test the hypothesis that health-related control beliefs predict recovery indices we report the outcome data taken from T3 (3 month follow up) for health behaviours, attendance at cardiac rehabilitation, depression, and QOL outcomes, and examine these associations using hierarchical linear regression models. Age, gender, BMI, income category, EuroSCORE, and depression symptoms, were entered into model 1, along with the baseline value of the relevant outcome variable. Demographics, clinical factors, measures of socio-economic status, as well as baseline depression were identified a priori as potential confounding variables as they have all been associated with lifestyle behaviour, adherence and QOL in coronary heart disease patients (Blumenthal et al., 2003; Burg et al., 2003). Health related personal control and treatment control were added in model 2 and 3 of the regression model to see if any additional variance would be explained over and above demographic and biological factors. Adjusted R^2^ and unstandardized beta values with 95 % confidence intervals are reported. Preliminary analyses were conducted to ensure that no violations of the assumptions of normality, linearity, multicollinearity, and homescedasticity.

## Results

A summary of demographic and clinical information can be seen in Table 1. The sample had an age range between 44 and 90 years, were predominantly male (89 %), and over-weight (BMI > 25 = 83 %). The majority of patients were hypertensive, and approximately a quarter were diabetic. Most patients had on-pump surgery. On average participants were within the normal range for depression symptoms on the BDI; however, 30 % of participants at baseline, and 21 % at follow up, scored >10. Overall, there were improvements in health outcomes for patients post-operatively assessed at 3 months. Paired *t* tests showed significant improvement from 1 month prior to surgery to 3 month post-operative follow up for depression symptoms, mental component QOL, and adherence (*p* < 0.05), with the exception a significant reduction in physical component QOL. Please see Table 2. Treatment control was not significantly associated with any of the outcome measures (*p* > 0.05) and so only the results for perceived personal control over cardiac illness are reported.

**Table 1:** Characteristics of the sample (N = 149)

Characteristic	Mean ± SD or N (%)
Age (years)	67.98 ± 8.23
Female	17 (11 %)
BMI (kg/m^2^)	28.37 ± 3.61
Ethnicity—White British	133 (89.3 %)
Yearly household income	
<10,000 GBP	16 (11 %)
10,000–20,000 GBP	42 (29 %)
20,000–30,000 GBP	38 (25 %)
30,000–40,000 GBP	24 (16 %)
>40,000 GBP	29 (19 %)
Logistic EuroSCORE(%)	4.15 ± 2.81
No. of grafts	3.00 ± 1.14
Length of hospital stay	7.00 ± 5.91

**Table 2:** Means and standard deviations of mood, QOL, and health behaviours pre and post CABG surgery

	Pre-surgery	Post-surgery (6–8 weeks)
	Mean (SD)	Mean (SD)
Depression symptoms	8.23 (5.73)	6.80 (6.34)*
Quality of life (PCS)	39.74 (11.02)	35.14 (8.02)*
Quality of life (MCS)	56.28 (6.56)	58.18 (6.65)*
Physical activity (hours per week)	3.39 (4.15)	3.51 (2.77)
Adherence to medication	1.48 (1.66)	1.02 (1.74)*

*PCS* physical component score, *MCS* mental component score

* A significant difference *p* < 0.05

### Health related personal control as a predictor of depression symptoms and QOL 6–8 weeks following surgery

#### Depression at 3-month follow up

We found that depressive symptoms at 3 months were significantly associated with baseline depression in model 1 (B = 0.513, *p* < 0.001, CI 0.354–0.672). Personal control was a significant independent predictor in model 2 (B = −0.376, CI −0.709 to −0.044, *p* = 0.027), and the variance accounted for significantly increased from 24.9 to 27.1 %.The results suggest that higher health related personal control was independently associated with lower depression levels 3 months later (see Table 3).

**Table 3:** Predictors of mood, QOL, and health behaviours 6–8 weeks following surgery

Model	Adj R^2^	F	B	95 % CI	*p*
Depression symptoms					
Model 1—covariates^a^	0.249	10.214			0.001
Model 2—personal control plus covariates^b^	0.271*	9.606			0.001
*Personal control*			−0.376	−0.709, −0.044	0.027
*Baseline depression*			0.513	0.354, 0.672	0.001
Physical component (QOL)					
Model 1—covariates^a^	0.009	1.265			0.283
Model 2—personal control plus covariates^b^	0.050*	2.219			0.045
*Personal Control*			0.652	0.155, 1.149	0.011
Mental component (QOL)					
Model 1—covariates^a^	0.082	3.476			0.006
Model 2—personal control plus covariates^b^	0.114*	3.983			0.001
* Personal Control*			0.460	0.085, 0.836	0.017
* Baseline Mental QOL*			0.289	0.130, 0.448	0.001
Physical activity					
Model 1—covariates^a^	0.168	6.610			0.001
Model 2—personal control plus covariates^b^	0.162	5.486			0.001
* Baseline walking hrs*			0.226	0.120, 0.332	0.001
* EurosSCORE*			−0.272	−0.441, −0.103	0.002
Adherence to medication					
Model 1—covariates^a^	0.182	6.145			0.001
Model 2—personal control plus covariates^b^	0.184	5.488			0.001
* Baseline adherence*			0.433	0.267, 0.599	0.001

* A significant increase in variance change

^a^Baseline adjustments: BMI, smoking status, income, euroSCORE, grafts, baseline depression, corresponding baseline values

^b^Adjusted model: personal control

#### Physical component (QOL) at 3-month follow up

The model 1 regression on the 3 month physical QOL ratings was not significant. However, in model 2 higher levels of health related personal control predicted better physical component QOL (B = 0.652, CI 0.155–1.149, *p* = 0.011). The variance accounted for in the final model was a small but significant increase (4.1 %), with personal control over cardiac illness the only independent predictor of outcome.

#### Mental component (QOL) at 3-month follow up

Baseline mental component (QOL) (B = 0.289, CI 0.130–0.448, *p* = 0.001) was the only significant predictor of mental component QOL in model 1, accounting for 8.2 % of the variance. There was a significant increase in variance rising to 11.4 % in model 2, health related personal control was significantly positively associated with mental component QOL (B = 0.460, CI 0.085–0.836, *p* = 0.017).

#### Health behaviours at 3-month follow up

Health related personal control was not associated with physical activity, adherence, or cardiac rehabilitation attendance at follow up (*p* > 0.05).

### Sensitivity analysis

Data were missing for one or more variables in 25.5 % (n = 55) of the total sample. Patients with missing data were more likely to be overweight (t = 2.06, *p* = 0.040), and have lower scores on the MoCA than those patients with complete data (t = 2.49, *p* = 0.014). No other differences were found on variables of age, gender distribution, marital status, diabetic status, EuroSCORE, or household income (*p* > 0.05). Furthermore, no differences were found for health-related personal control or treatment control variables. Nor on outcome measures of QOL, depression symptoms, adherence, physical activity, or cardiac rehabilitation attendance (*p* > 0.05).This suggests that participants with missing data did not always have unfavourable risk factors or outcomes.

In order to account for any potential bias due to missing data we re-ran the analysis for all participants. This did not change the original findings; for example, treatment control did not predict any outcome measure, and personal control remained a significant predictor of depression symptoms (B = −0.347, CI −0.618 to −0.075, *p* = 0.013), mental component QOL (B = 0.351, CI 0.042–0.660, *p* = 0.028), and physical component QOL only (B = 0.495, CI 0.059–0.931, *p* = 0.026).

## Discussion

The purpose of this study was to evaluate whether health-related control beliefs predicted depression symptoms, QOL, and health behaviours 6–8 weeks following CABG surgery. Patients showed significant improvements at 3-month follow up in depression symptoms, mental component QOL, and adherence behaviour; in contrast, physical component QOL was significantly reduced. Consistent with our hypotheses we found that low perceived personal control over their cardiac illness predicted elevated levels of depression symptoms, and poorer physical and mental QOL, independently of covariates. In contradiction to our predictions, we found no association for health-related personal control and adherence to medication, physical activity, or attendance at cardiac rehabilitation. Perceived treatment control was not associated with any outcome measure.

The results from our study suggest that lower perceived personal control over their cardiac illness predicts elevated levels of post-operative depression symptoms in CABG patients. This remained the case even after controlling for demographic, clinical, and pre-operative depression variables in the analyses. Our findings are in line with the extensive literature detailing the association between low perceived control and elevated depression in other groups of cardiac patients (Dracup et al., 2003; Foxwell et al., 2013). The prevalence of high levels of depression symptomatology (30 %) in this CABG population is consistent with levels reported in the research literature (Burg et al., 2003).

Previous research has established that there is a strong association between psychological distress and poor health outcomes following CABG (Blumenthal et al., 2003). Indeed, depression is viewed as an independent contributor to the development and progression of cardiac problems after CABG (Burg et al., 2003). Moreover, depression is not only an adverse outcome in its own right, but is often implicated with reduced QOL, and may be indirectly associated with recovery via poorer health behaviours and adherence to medical regimes (Doering et al., 2005; Oxlad & Wade, 2008). Therefore, identifying risk factors that are amenable to change, is important in the treatment of CHD (Moser et al., 2007).

In line with the existing research, we found that pre-operative depression predicted mental component QOL, but not physical QOL outcomes at 3 months (Oxlad & Wade, 2008). Having low perceived personal control, however, independently predicted both reduced physical and mental QOL outcomes 3 months post-operatively. Previous research has identified a beneficial effect of health related perceived control on physical functioning and QOL outcomes up to 6 months following CABG surgery (Barry et al., 2006; Duits et al., 1997).

Neither treatment control nor personal control was associated with adherence to medication, attendance at cardiac rehabilitation, or physical activity. Several explanations can be offered for these results. There may be little or no associations between health-related control beliefs and health behaviours in CABG patients (Byrne et al., 2005), though this seems unlikely given work in other cardiac populations (French et al., 2006). Alternatively, the results may have been influenced by age, since Gump et al. (2001) found that older patients were less likely to change their health behaviour post-operatively.

Methodological factors may be responsible for the inconsistency in findings. Firstly, using a single item to measure treatment control or perceived personal control may not give a true reflection of relatively complex constructs. Secondly, using a complete case approach for data analysis may lead to bias in the results. Thirdly, looking at health behaviour as short-term outcomes during the recovery period may be responsible for the non-significant findings. Typically, during the first few months following surgery patients will experience some degree of physical restriction. Physical problems after CABG surgery can include pain from chest and leg incisions; wound infection, loss of appetite, and fatigue (Gallagher et al., 2004). It can take up to 4 months for patients to be able to resume normal daily activities such as climbing the stairs, walking, gardening, and driving (Wilson-Barnett, 1981). Recovery is also facilitated by the care received from family members or friends, who help with food shopping, food preparation, medication reminders, and accompany the patient to hospital appointments (Theobald et al., 2005). Until they recover fully and are able to be self-reliant, treatment control and personal control may be less pertinent than other factors to health behaviours in the shorter term.

Currently little is known regarding the mechanisms that may underlie the associations between control and recovery following CABG surgery. Patients with low control may experience feelings of increased stress and helplessness, which have been linked to negative moods and behavioural outcomes (Lachman et al., 2010; Seeman & Seeman, 1983).There is also substantial evidence that lack of control has adverse effects on biological processes relevant to health, including cardiovascular activity, neuroendocrine responses, and immune processes (Steptoe & Appels, 1989). It is clear that more research is needed to address how these processes operate and influence recovery in CABG patients.

In spite of these limitations, our study has a number of strengths. We recruited a relatively large prospective sample of elective CABG patients, and obtained multiple measures regarding health behaviours, QOL, depression symptoms and illness perceptions prior to and 6–8 weeks following surgery. Our findings extend the existing literature in two ways. Firstly, we have demonstrated that health related personal control predicts depressive symptoms and QOL 6–8 weeks after surgery, over and above traditional risk factors (Juergens et al., 2010), and that single illness perception items can be used prospectively to predict health outcomes (Petrie & Weinman, 2012).

Second, existing studies have shown that challenging maladaptive illness perceptions following a cardiac event, such as an MI, can result in impressive improvements in specific recovery outcomes such as return to work (Broadbent et al., 2009). Attempts have also been made to enhance patient expectations prior to surgery in order to improve recovery (Laferton et al., 2015). Our results not only support this proposition but also suggest interventions that focus on perceived personal control over cardiac illness might maximise short-term recovery outcomes. Future work should address the efficacy of such a proposal in both short and longer-term recovery outcomes in CABG patients.
